# Dose-response relationship for breast cancer induction at radiotherapy dose

**DOI:** 10.1186/1748-717X-6-67

**Published:** 2011-06-08

**Authors:** Uwe Schneider, Marcin Sumila, Judith Robotka, Günther Gruber, Andreas Mack, Jürgen Besserer

**Affiliations:** 1 Vetsuisse Faculty, University of Zürich, Winterthurerstrasse 260, 8057 Zürich, Switzerland; 2 Institute for Radiotherapy, Hirslanden Hospital Zürich, Witellikerstrasse 40, 8032 Zürich, Switzerland

**Keywords:** second cancer, breast cancer, carcinogenesis

## Abstract

**Purpose:**

Cancer induction after radiation therapy is known as a severe side effect. It is therefore of interest to predict the probability of second cancer appearance for the patient to be treated including breast cancer.

**Materials and methods:**

In this work a dose-response relationship for breast cancer is derived based on

(i) the analysis of breast cancer induction after Hodgkin's disease,

(ii) a cancer risk model developed for high doses including fractionation based on the linear quadratic model, and

(iii) the reconstruction of treatment plans for Hodgkin's patients treated with radiotherapy,

(iv) the breast cancer induction of the A-bomb survivor data.

**Results:**

The fitted model parameters for an *α/β = 3 *Gy were *α = 0.067Gy*^*-1 *^and *R = 0.62*. The risk for breast cancer is according to this model for small doses consistent with the finding of the A-bomb survivors, has a maximum at doses of around 20 Gy and drops off only slightly at larger doses. The predicted *EAR *for breast cancer after radiotherapy of Hodgkin's disease is 11.7/10000PY which can be compared to the findings of several epidemiological studies where EAR for breast cancer varies between 10.5 and 29.4/10000PY. The model was used to predict the impact of the reduction of radiation volume on breast cancer risk. It was estimated that mantle field irradiation is associated with a 3.2-fold increased risk compared with mediastinal irradiation alone, which is in agreement with a published value of 2.7. It was also shown that the modelled age dependency of breast cancer risk is in satisfying agreement with published data.

**Conclusions:**

The dose-response relationship obtained in this report can be used for the prediction of radiation induced secondary breast cancer of radiotherapy patients.

## Background

Cancer induction after radiation therapy is known as a severe side effect. It is therefore of interest to predict the probability of second cancer appearance for the patient to be treated. For this purpose it is not sufficient to apply the results from epidemiological studies on cancer induction from more than 20 years ago to the patient treated today, since radiation therapy changed significantly in the last decades, for instance radiation type, treatment technique, application of treatment, treatment duration and 3D dose distributions.

As a consequence it is necessary to model cancer induction for patients undergoing radiotherapy and thus the underlying dose-response relationship [1-3]. Such modelling can be based on epidemiological studies of patients treated with old techniques. However, most of the epidemiological studies, which are published in large numbers, don't provide a correlation of cancer induction with dose. Unfortunately, if a dose correlation is deduced, cancer induction is usually related to the integral dose or average organ dose and thus implies a linear dose-response relationship. Therefore, such data cannot be used directly to obtain non-linear dose-response relationships. Up to now there are only few studies which correlate cancer induction in radiotherapy patients with point dose estimates at the location of secondary tumor growth [4-10].

Radiotherapy of patients with Hodgkin's disease is very successful, but women treated with mantle field radiation experience up to a 30-fold increased risks for breast cancer compared with their peers in the general population. Travis et al [8] for instance studied breast cancer induction for mantle field treatments of Hodgkin's disease. They reconstructed the point doses where the secondary breast cancer was located and performed a case/control study to stratify breast cancer risk as a function of dose.

The goal of this report is the derivation of a dose-response relationship for breast cancer induction based on the analysis of Hodgkin's disease patients by Travis et al [8] and breast cancer induction from the A-bomb survivors [11]. A recently developed cancer induction model [12] including fractionation was fitted to the available data. The model was tested by predicting second cancer risk resulting from historical mantle field treatments for Hodgkin's disease and comparing them to published epidemiological data. In addition model predictions were compared to recently published second breast cancer risk for mediastinal involved field radiotherapy.

## Materials and methods

### Dose-response model

It is assumed that cancer induction is proportional to the number of cells in the tissue and thus to the mass of the tissue. Since we are analyzing breast tissue only, cancer induction is considered to be proportional to the involved volume assuming a constant cell density over the whole breast. The tissue is irradiated with a fractionated treatment schedule of equal dose fractions *d *up to a dose *D*. The number of original cells after irradiation is reduced by cell kill which is proportional to *α' *and is defined using the linear quadratic model(1)

It is further assumed for this work, that the number of killed original tissue cells is replaced by a number of new cells. Additionally it is assumed that the repopulation kinetics of repopulated cells will follow the same basic patterns as those of normal cells. Cells which were irradiated can be mutated and have the potential to develop a tumor. In the context of this work the word "mutation" is used as a synonym for each cell transformation which develops new tumor cells. In fact the development of a tumor usually implies several mutations. The mutational process for one dose fraction is modelled according to the linear-no-threshold model and thus cancer risk originating from an irradiation with one dose fraction *d *is taken proportional to *μ *which is the slope of cancer induction from the linear-no-threshold model which is mainly based on the data of the A-bomb survivors. It is finally assumed that the number of involved cells is treated as a continuous function of dose, a system of differential equations derived from the cell kinetics can be solved [12]. The excess absolute risk for carcinoma induction is then(2)

where *R *is the fraction of repopulated cells at the end of treatment and thus characterizes the ability of the tissue to repopulate. Tissue which is not able to repopulate/repair corresponds to *R = 0 *and complete repopulation/repair is characterized by *R = 1*. Eq. 2 was obtained from [12] by substituting *R = ξ(α'+ ξ) *into Eq. 7a of [12] where *ξ *was originally introduced to describe the repopulation/repair rate. Risk equivalent dose (*RED*), as defined by Eq. 2, is a dose-response weighted local dose value which is by definition proportional to risk. When *RED *is averaged over the whole breast the organ equivalent dose (OED) can be calculated [1]. OED which is measured in Gy is then directly proportional to cancer risk in the breast:(3)

where the sum is taken over all volume elements *V*_*i *_of the breast and *V*_*Breast *_is the total breast volume.

It is assumed here an *α/β *= 3 Gy for breast tissue. However, *α/β *= 1 Gy and *α/β *= 5 Gy were also used for optimization to test the robustness of the model.

A requirement for any realistic dose-response model is that the predicted cancer risk approaches in the limit of low dose the well known linear-no-threshold (LNT) model which is usually used for risk estimates in radiation protection. The excess absolute risk for breast cancer induction at low dose derived from the A-bomb survivor data according to Table 29 in Preston et al [11] is 9.2 (CI95: 6.8-12) cases per 10000 persons per year per Gy at age 70 after exposure at age 30. This value must be modified to fit the age distribution of the cohort of the Travis [8] study. Average age at diagnosis (agex) of the Hodgkin's disease patients was 22 years. The patients developed breast cancer in average 18 years after diagnosis of Hodgkin's disease, which results in an attained age (agea) of 40. The LNT-risk for breast cancer induction is according to [11]:(4)

where the age modelling was centered around 30 and 70 years, respectively. This risk representing the A-bomb survivor data is plotted with the corresponding error bar in all figures of this report as a dashed line.

### Patient data and statistical analysis

In the analysis for this work a matched case-control study conducted by Travis et al [8] was used. The study analysed a population-based cohort of 3817 women who were treated for Hodgkin's disease between 1965 and 1994. The mean and median age at diagnosis was 22 years. Point dose reconstruction for the breast cancer was possible for 102 cases and 257 controls. Patients with breast cancer were grouped into 7 dose categories (Table 1).

**Table 1:** Point dose estimates and related odd ratios for breast cancer after radiotherapy of Hodgkin's disease from Travis et al [8].

Median dose (range)[Gy]	Cases	Controls	Odds ratio (stand. dev.)	p-value	EAR optimized with A-bomb *agex *= 30 *agea *= 70, *α/β *= 3 (std. dev.)
3.2 (0-3.9)	15	76	Reference	Reference	19.3
4.6 (4.0-6.9)	13	30	2.2 (1.4-3.4)	0.07	42.5 (27.5-65.7)
21.0 (7.0-23.1)	16	30	2.7 (1.8-4.1)	0.02	52.3 (34.4-79.5)
24.5 (23.2-27.9)	9	30	1.5 (0.9-2.4)	0.38	29.4 (18.3-47.2)
35.2 (28.0-37.1)	20	31	3.3 (2.2-4.9)	<0.01	63.3 (42.3-94.6)
39.8 (37.2-40.4)	12	31	2.0 (1.3-3.1)	0.13	38.0 (24.4-59.0)
41.7 (40.5-61.3)	17	29	3.0 (2.0-4.5)	0.01	57.5 (37.9-87.1)

EAR was optimized for age at exposure of 30 years, attained age 70 years and α/β = 3Gy.

The unadjusted odds ratio was computed from controls and cases, and the error factor and confidence levels were obtained using maximum likelihood estimates. The odds ratio, which approximates relative risk, is listed in Table 1.

The model parameters *α *and *R *of Eq.2 were optimized by a variation in the interval [0,1] for both case-control studies independently. For any combination of (α_, _*R*)∈ [0,1] the relative risks of Travis et al [8] were converted to excess absolute risk. The risk for radiation induced cancer after radiation therapy is better modelled using excess absolute risk (EAR) as expressed by Eq. 2, since relative risk estimates make only sense when patients with the same dose distributions are compared and this is most often not the case for radiotherapy patients. As EAR defined by Eq. 2 approaches for small dose the LNT model it was assumed that the risk of the lowest dose category corresponds to the findings of the A-bomb survivor data. This correspondence was used to transform the Travis data, expressed in odds ratios, into EAR. However, the LNT risk for breast cancer (*μ *= 4.8/10000PY/Gy according to Eq. 4) is subject to an uncertainty between 3.5 and 6.2/10000PY/Gy (95% CI-interval according to [11]). This uncertainty was included in the model fit for the lowest dose category.

The model parameters *α *and *R *were determined by a least square minimization of(5)

The parameters were optimized using a 0.1% precision criteria and were performed for three different *α/β *values (1, 3, 5 Gy). The standard deviation of the fitted parameters were calculated from the error of the odds ratios by Gaussian error propagation using the partial derivatives of Eq. 2 and are listed in Table 2. It was further assumed that the total number of person years in the seven dose groups is comparable.

**Table 2:** Fitted model parameters with the corresponding standard deviation for different α/β-values.

Fitted parameter	α/β [Gy]		
	**1**	**3**	**5**
α (±σ_α_)/[Gy^-1^]	0.036 (0.021-0.076)	0.067 (0.033-0.112 )	0.080 (0.042-0.130)
R (±σ_R_)	0.66 (0.43-0.92)	0.62 (0.34-0.90)	0.62 (0.34-0.90)

### Dose reconstruction for risk predictions

Dose distributions were reconstructed, which were characteristic for a large patient collective of Hodgkin's disease patients. We calculated the dose distributions in an Alderson Rando Phantom with a 200 ml breast attachment.

Typical treatment techniques for Hodgkin's disease radiotherapy were reconstructed. Treatment planning was performed on the basis of the review by Hoppe [13] and the German Hodgkin disease study protocols (http://www.ghsg.org). We used for treatment planning the Eclipse External Beam Planning system version 8.6 (Varian Oncology Systems, Palo Alto, CA) using the AAA-algorithm (version 8.6.14). Treatment plans were computed which included mantle field treatment and treatment of supraclavicular, axillary and mediastinal lymph nodes for both, left and right location. All plans were calculated with 6 MV photons and consisted of two opposed fields. The technique for shaping large fields included divergent lead blocks. Treatment was performed at a distance of 100 cm (SSD). Anterior-posterior (ap/pa) opposed field treatment techniques were applied to insure dose homogeneity.

The mantle field included the bilateral cervical, supraclavicular, axillary, infraclavicular, mediastinal and pulmonary hilar lymph nodes. The unblocked field size was 34 cm × 33 cm with equal field weights from 0° and 180°. The superior border of the mantle was located along the base of the mandible, and the inferior border was at the level of the insertion of the diaphragm (T10 vertebra). Blocks were placed over the lung and the humeral heads both anteriorly and posteriorly. Spinal cord blocking was not needed, since the planned total dose was 38 Gy, which is the average dose of the patients studied by Travis et al [8]. All blocks were contoured by hand.

The pelvic field included bilateral iliac and inguinal lymph nodes with 2 cm safety margins laterally. The superior border was drawn at the L4-5 interspace, the inferior border was bilateral at the inferior border of the obturatorial foramen.

The supraclavicular field included the ipsilateral supraclavicular fossa and the lower cervical lymph nodes, that means from the inferior border of the hyoid bone to 1.5 - 2 cm below the clavicle.

The axillarv field encompassed the axillar lymph nodes. It included the periclavicular region and reached caudally to the 6th rib. A small peripheral lung zone of 1.5 cm was included. We used a block over the humeral head. The mediastinal field included both the superior and inferior mediastinal and hilar lymph nodes in addition to the lower cervical and supraclavicular lymph nodes (medial 2/3 of clavicula). The upper border was the hyoid bone, the lower border the insertion of the diaphragm. The field border was on each site 1.5 cm inferior to the clavicule, along transversal processi and 1.5 cm laterally from each hilus.

## Results

### Results of the model fit

**Figure 1:**
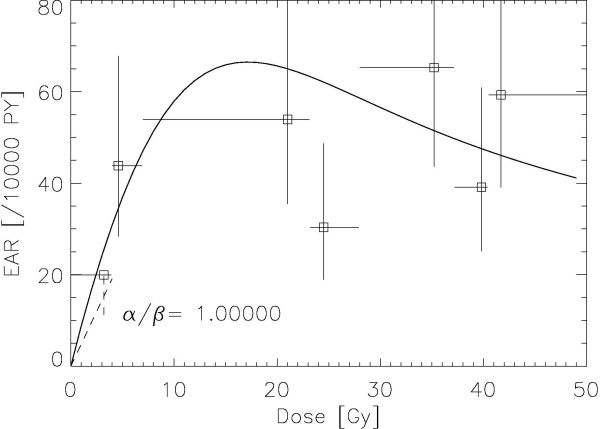
**Plot of the modelled excess absolute risk (solid line) to the epidemiological data of Travis et al **[8]**for α/β = 1 Gy**. The dashed line represents the LNT-model for breast cancer with the corresponding error [10].

**Figure 2:**
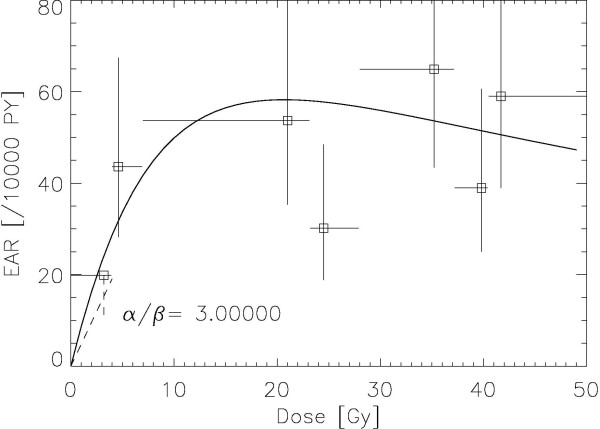
**Plot of the modelled excess absolute risk (solid line) to the epidemiological data of Travis et al **[8]**for α/β = 3 Gy**. The dashed line represents the LNT-model for breast cancer with the corresponding error [10].

**Figure 3:**
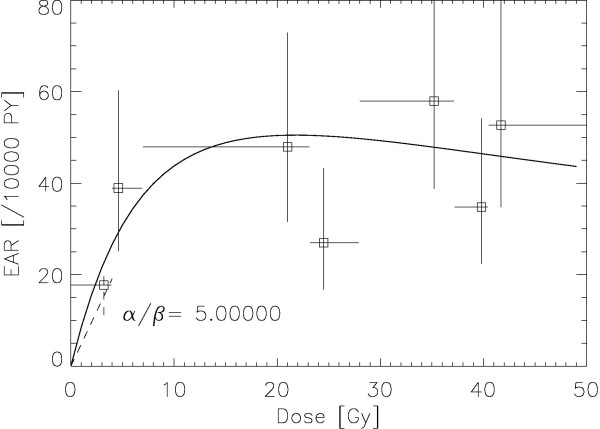
**Plot of the modelled excess absolute risk (solid line) to the epidemiological data of Travis et al **[8]**for α/β = 5 Gy**. The dashed line represents the LNT-model for breast cancer with the corresponding error [10].

The results of the fitting procedure to the Travis data [8] are displayed for *α/β *= 1, 3, 5 Gy in Figures. 1, 2 and 3, respectively. The squares represent the data points from the work of Travis et al. [8] for the outlined breast volume with the corresponding dose (one standard deviation). Modelled risk is the average of the left and right breast. It should be noted here that the dose axis shows the total dose in breast tissue after the end of treatment and not the cumulated target dose. The optimized model parameters are listed in table 2 for *α/β *= 1, 3 and 5 Gy. A variation of *α/β *from 1 Gy to 5 Gy shows no significant differences in breast cancer risk at high dose.

### Comparison of modelled breast cancer risk with published results of mantle field treatment

The dose-response relationship for breast cancer induction obtained in this work was used to predict female breast cancer risk resulting from independent epidemiological studies of mantle field treatments of Hodgkin's disease.

Data for female breast cancer risk were taken from the publications of Hancock and Hoppe [14] who found an *EAR*_*Breast *_*= 21.5/10000PY*, from Swerdlow et al [15] 3.1*/10000 PY*, from Dores et al [16] 10.5*/10000PY *and from van Leeuwen et al *29.4/10000PY *[17]. The mean age at exposure and attained age of the respective patient cohorts are listed in Table 3 and were used for the model calculations with Eq. 4. Calculations using the model parameters with *α/β *= 3 Gy resulted in an *EAR *of *10.6/10000PY*, *11.7/10000PY*, *11.0/10000PY *and *12.9/10000PY *for Hancock and Hoppe, Swerdlow, Dores and van Leeuwen, respectively and are listed in Table 4. These predictions can be viewed as a test of the model.

**Table 3:** Cohort size (number of patients), median age at exposure and attained age for the published breast cancer rates after Hodgkin's disease radiotherapy.

Published breast cancer risk after Hodgkin's disease	Cohort size	Age at exposure	Age at exposure + mean follow-up
Dores et al. [16]	32'591	37	45
Hancock and Hoppe [14]	2'162	29	40
Swerdlow et al. [15]	5'519	36	45
van Leeuwen et al. [17]	1'253	24	38

**Table 4:** Modelled breast cancer risk for different α/β-values for mantle field treatment of Hodgkin's disease and comparison with published data.

EAR [/10000 PY]	**Dores et al **[16]	**Hancock and Hoppe **[14]	**Swerdlow et al **[15]	**van Leeuwen **[17]	average
observed	10.5	21.5	3.1	29.4	16.1

α/β = 1 Gy	12.0 (10.9-13.7)	13.2 (12.0-15.0)	12.4 (9.1-15.1)	14.5 (13.2-16.6)	13.0

α/β = 3 Gy	10.7 (8.3-14.3)	11.8 (9.2 -15.8)	11.1 (8.7-14.9)	13.0 (10.1-17.4)	11.7

α/β = 5 Gy	10.3 (8.0-13.7)	11.3 (8.1-13.7)	10.7 (8.4-14.2)	12.5 (9.8-16.6)	11.2

It should be noted here that the statistical power of the published data is quite different due to the different cohort sizes (Table 3) involved. The data from Dores et al [16] are by far the most reliable since the number of observed persons is six-times larger than the second largest group.

### Comparison of modelled breast cancer risk with published results for involved field treatment

De Bruin et al [18] recently assessed the long-term risk of breast cancer after treatment for Hodgkin's lymphoma. In contrast to other researchers they focused on the risk after smaller radiation volumes. De Bruin et al [18] performed a cohort study among 1,122 female 5-year survivors treated for Hodgkin's lymphoma and compared the incidence of breast cancer with that in the general population. During follow-up, 122 patients developed breast cancer. All of them had previously received radiotherapy with a dose of 40 Gy (36 to 44 Gy) in fractions of 2.0 Gy. The median follow-up time for the total cohort was 17.8 years. The median age at first treatment for Hodgkin's lymphoma was 26.3 years. The distribution of radiation fields was carefully recorded and is listed in Table 5 together with the treatment techniques for which De Bruin et al determined risk.

**Table 5:** Comparison of modelled and observed relative breast cancer risk for involved field radiotherapy.

Technique	Used Treament plans	Weighting according to # treated patients	Relative OED (Travis fit)	Observed relative risk
Mediastinal	Mediastinal	109	1	1

Mantle	Mantle field alone	637	3.2	2.7 (1.1-6.9)

other Supradiaphragmatic	Supraclavicular/neck	34		
	Axillary + Mediastinal/homolat	41		
	Axillary + Mediastinal/bilat	7		
	Axillary, no Media.	14		
	Total	96	1.9	0.9 (0.2-4.8)

Modelling was performed forα/β = 3Gy. Since OED is proportional to risk relative OED it can be compared to observed relative risk.

Breast cancer risk for the cohort analysed by De Bruin et al [18] was modelled using the dose-volume histograms for the left and right breast obtained from the treatment plans listed in Table 5. OED was calculated using Eqs. 2-4 with an *α/β *= 3 Gy using the fitted model parameters from Table 2. Since OED is additive the total OED for a treatment technique was determined using the weighting of the treatment fields of Table 5.

### Comparison of modelled age dependence of breast cancer risk with clinical results

**Figure 4:**
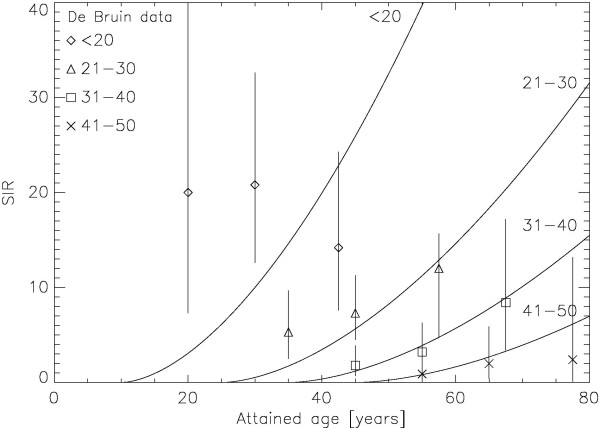
**Plot of the modelled age dependence of the standardized incidence ratio (normalised to the De Bruin data) as the solid lines for the age at treatment groups <20, 21-30, 31-40 and 41-50, respectively**. The corresponding epidemiological data from De Bruin are plotted as the symbols together with the corresponding 95% confidence interval.

Another question is whether the age dependence of breast cancer of the presented model which is based on the recent data of the A-bomb survivors fits clinical data of breast cancer induction after radiotherapy. For this purpose the modelled age dependence according to Eq. 4 was compared to the published results of De Bruin et al [Table 3 in 18]. In Figure 4 the modelled age dependence of risk, normalised to the De Bruin data, is shown together with the corresponding epidemiological data from De Bruin as the symbols. The model agrees well for the age groups 21-50. The age group <20 years shows significant differences. The involved errors, however, are large.

## Discussion

The aim of this study was the determination of model parameters for a dose-response relationship for breast cancer covering dose levels relevant for radiotherapy. In addition a model for the age dependence of breast cancer risk was verified. The model was tested with epidemiological data on second breast cancer of historic mantle field treatments and high dose involved field radiotherapy. Satisfying agreement was found. In the limit of small dose the model approaches the LNT-model for cancer induction.

In this report a cancer induction model for the radiotherapy dose range was used. Several assumptions had to be made to simplify the biological processes leading to cancer induction [12]. This includes the design of tissues, the repopulation process and processes which result in the formation of a tumor cell. This was done to keep the number of model parameters at a minimum. However, this is associated with uncertainties.

When interpreting the results of this study, certain limitations should be taken into account. The model was fitted to epidemiological data describing breast cancer risk after radiotherapy of Hodgkin's disease. Several assumptions were made to use these data for model fitting. It has been hypothesized that the age parameters of the complete patient cohorts can be applied to the patients grouped in different dose categories. In addition the median/averages of the characteristic age parameters were used knowing that the ages can vary significantly and that the age dependence is in general non-linear.

In addition the impact of ovarian function on breast cancer induction is not included in the model. Chemotherapy and pelvic radiotherapy could have a protective effect regarding breast cancer induction. However, in the publication of De Bruin et al [18] such an effect was not found.

In this work EAR has been used to quantify radiation-induced cancer. Usually excess relative risk (ERR) is recommended for transferring risk from the Japanese population to other populations. EAR is used here, since the risk calculations of the Hodgkin's cohort are based on extremely inhomogeneous dose distributions. Currently there is no method available for obtaining analogous organ risks using ERR. As the difference between the Japanese and the US population in EAR for all solid tumors is less than 10% the use of EAR is probably justifiable.

Additionally, as the results of this report are expressed in terms of EAR, it is also difficult to compare them with the findings of Sachs and Brenner [2] who fitted an algebraic model of cancer induction to breast cancer risk. The risk ratio between historic mantle field treatments and high dose involved field radiotherapy is however comparable with other ERR models [19].

The treatment plans calculated in this work were computed using 6 MV photons. Apparently, patients treated in a time period of nearly 30 years were irradiated with x-ray beams of various energies. Since De Bruin et al [18] presented no information on the range of treatment energies, it was decided to use 6 MV photons. However, this could have an impact on the calculated dose distributions in particular on the deposited energy from scattered radiation.

## Conclusion

In this work a dose-response relationship for breast cancer was derived based on the analysis of breast cancer induction after Hodgkin's disease, a cancer risk model developed for high doses including fractionation based on the linear quadratic model, and the reconstruction of treatment plans for Hodgkin's patients treated with radiotherapy.

The fitted model parameters for an *α/β *= 3 Gy and *μ *= 4.8/10000PY/Gy were α = 0.067 Gy^-1 ^and *R *= 0.62. Breast cancer risk is according to this model for small doses consistent with the findings of the A-bomb survivors, has a maximum at doses of around 20 Gy and drops off only slightly at larger doses. The predicted EAR for breast cancer after radiotherapy of Hodgkin's disease is 11.7/10000PY which can be compared to the findings of several epidemiological studies were EAR for breast varies between 10.5 and 29.4/10000PY. The model was used to predict the impact of the reduction of radiation volume on breast cancer risk. It was predicted that mantle field irradiation is associated with a 3.2-fold increased risk compared with mediastinal irradiation alone. This is comparable to the findings of De Bruin et al [18] who found a 2.7-fold increase.

It was also shown that the modelled age dependency of breast cancer risk based on the A-bomb survivor data is in satisfying agreement with published data on breast cancer risk after radiotherapy of Hodgkin's disease. The work presented here might provide the first direct evidence that cancer risk age modelling based on the A-bomb survivor data can be applied to radiotherapy patients.

The dose-response relationship obtained in this report can be used for the prediction of radiation induced secondary breast cancer of radiotherapy patients. It might be used to further optimize radiation therapy of Hodgkin's disease with regard to second breast cancer. In addition the obtained *α*-value for breast tissue can be used for applications of the linear-quadratic model in radiotherapy.

## Competing interests

The authors declare that they have no competing interests.

## Authors' contributions

US designed this study, performed the modelling, and drafted the manuscript. MS and JR performed the treatment planning and the dose reconstruction for the risk predictions. JB, AM and GG participated in the risk predictions. All authors read and approved the final manuscript.
